# P234: Acinetobacter infections in intensive care units in 2011

**DOI:** 10.1186/2047-2994-2-S1-P234

**Published:** 2013-06-20

**Authors:** H Gül, A Karakaş, C Artuk, G Mert, S Akyüz, CP Eyigün

**Affiliations:** 1 Infectious Diseases and Clinical Microbiology, Gülhane Military Hospital, Ankara, Turkey; 2 Infection Control, Gülhane Military Hospital, Ankara, Turkey

## Introduction

Mortality rate of *Acinetobacter* infections have increased due to having multiple drug resistance and increasing of incidence among nosocomial infection. Importance of high mortality rate of this infections is once more increased due to scantiness of choosing antibiotics of treatment and difficulty of isolation.

## Objectives

Distribution of *Acinetobacter* spp which were identified in intensive care units (ICUs) and antimicrobial susceptibility patterns of them have been investigated in this study.

## Methods

*Acinetobacter* infections which were isolated in ten ICUs of GMMA in 2011 have been investigated retrospectively.

## Results

69 *Acinetobacter* strains were isolated from samples of 64 patients who were hospitalized in ICUs. *Acinetobacter* infections have became two times in five patients. 21 of patients were female, 43 of them were male and average age was 61,09±24,16 (2-91) years. Distributions of *Acinetobacter* infectious of ICUs were investigated and the most infectious (49,3% n:34) were occured at medical ICU followed by anesthesia recovery unit(20,3% n:14), burn unit (8,7% n:6), ICU of general surgery unit(7,2% n:5), ICU of neurology unit (5,8% n:4), respectively. The avarage of interval between hospitalization date and devolopment time of infectious was 27,78±40,02 (3-282) days and it varies according to the different ICUs Bloodstream infection was the most common infection, followed by urinary tract infection, pneumonia (n:10 14,4%), skin and soft tissue infections (n:8 11,6%), respectively. The most common species was *A. baumanni* (n:45 65,2%) and *A. calcoaceticus* (n:3 4,3%) was the second. When antimicrobial susceptibility patterns of isolated acinetobacter spp were investigated, susceptibility rates were determined as 100% to colistin, 27,2% to tigecycline, 25% to amikacin, 19,1% to gentamicin, 18,4% to cotrimoxazole, 5,1% to ciprofloxacin, 4,4% to piperacillin-tazobactam, 3,3% to aztreonam, 3,2% to cefotaxime, 3,1% to ceftazidime, 1,4% to imipenem and meropenem.

## Conclusion

Number of *Acinetobacter* infections are increasing corralated with the number of beds in ICUs. Isolation of infected patients and obeying the rules of universal contact isolation of health care personnel are important. Resistance rate of tigecycline was increased in short time after tigecycline began usage.

## Disclosure of interest

None declared

